# Trp64Arg Polymorphism in Beta3-Adrenergic Receptor Gene Is Associated with Decreased Fat Oxidation Both in Resting and Aerobic Exercise in the Japanese Male

**DOI:** 10.1155/2009/605139

**Published:** 2009-12-22

**Authors:** Emiko Morita, Hiroshi Taniguchi, Motoyoshi Sakaue

**Affiliations:** ^1^Graduate School of Human Science and Environment, University of Hyogo, 1-1-12 Shinzaike-Honmachi, Himeji, Hyogo 670-0092, Japan; ^2^Department of Physical Therapy, Faculty of Nursing and Rehabilitation, Aino University, 4-5-4 Higashiohda, Ibaraki, Osaka 567-0012, Japan; ^3^Yamato Institute of Lifestyle-Related Diseases, 5-22 Saenba-Cho, Akashi, Hyogo 673-8688, Japan

## Abstract

The purpose of our study was to investigate whether the Trp64Arg polymorphism in *β*3-AR gene and the −3826A/G polymorphism in the UCP1 gene were associated with the reduction in energy expenditure and fat oxidation both in resting and aerobic exercise in Japanese. Eighty-six nonobese young healthy Japanese were recruited. Energy expenditure was measured using indirect calorimetry. The subjects performed an aerobic exercise program at 60% of their maximal heart rate for 30 minutes. The level of fat oxidation at rest and aerobic exercise of the male subjects with Trp/Arg of the *β*3-AR gene was significantly lower than that of the Trp/Trp genotype. No difference in FO_0−30_ was observed in the female subjects. There was no association between UCP-1 polymorphism and energy expenditure during aerobic exercise. It was revealed that the Trp64Arg polymorphism in *β*3-AR gene is associated with reduction of fat oxidation both in resting and aerobic exercise in healthy, young Japanese males.

## 1. Introduction

Aerobic exercise is one of the major strategies in the prevention and the treatment of obesity and type 2 diabetes [1]. It is well known that aerobic exercise increases not only energy expenditure but also glucose uptake of the muscle cells by translocating the glucose-transporter from the cytoplasm to the cell surface, leading to lowering plasma glucose level and attenuating insulin resistance [2]. 

The effects of aerobic exercise on weight reduction vary among individuals. It is influenced by a variety of factors such as environmental factors, exercise intensity, muscle mass, level of circulating hormones, age, and gender. Genotypes of the genes involved in energy expenditure also affect efficiency of the aerobic exercise, but it remains unclear whether the strength and the length of the aerobic exercise should be modified by the genotypes of each patient with diabetes. 

*β*3-Adrenergic receptor (*β*3-AR) and uncoupling protein 1 (UCP1) are involved in energy expenditure of the adipocytes, and the polymorphism of these genes has been reported to be associated with the prevalence of obesity and type 2 diabetes. The prevalence of Japanese with Trp to Arg substitution at codon 64 of the *β*3-AR gene was documented to be relatively high and associated with early onset of type 2 diabetes [3]. This polymorphism was also reported to be associated with abdominal obesity, BMI, and insulin resistance [4–6]. Uncoupling protein 1 (UCP1), which is predominantly expressed in the brown adipose tissue, also plays an important role in energy homeostasis [7]. UCP1 alters respiration coupling and dissipates oxidation energy as heat maintaining body temperature [8]. From recent studies in humans, it is thought that the −3826A/G polymorphism in the UCP-1 gene is associated with vulnerability to weight gain and higher BMI [9]. In addition, there are several reports demonstrating that these two polymorphisms act synergically to lower the basal metabolic rate [10, 11]. 

Although these polymorphisms were demonstrated to be associated with expenditure at rest, few studies have shown the association of these polymorphisms with the energy expenditure during exercise. The purpose of our study was to investigate whether the energy expenditure and the fat oxidation during aerobic exercise were affected by the presence of mutant alleles in these genes.

## 2. Subjects and Methods

### 2.1. Subjects

Japanese college students were recruited for this study. This study was approved by the Aino University ethics committees. It conformed to the principles outlined in the Helsinki Declaration, and written informed consent was obtained from all participants before entry into this study.

### 2.2. Genotyping of *β*3-AR and UCP1 Genes

Genomic DNA was extracted from peripheral blood cells using a GFX Genomic Blood DNA Purification Kit (GE Healthcare, UK). The Trp64Arg polymorphism in the *β*3-AR gene and the −3826A/G polymorphism in the UCP1 gene were genotyped by polymerase chain reaction—restriction fragment length polymorphism analysis [12, 13].

### 2.3. Energy Expenditure, Fat Oxidation, and Carbohydrate Oxidation

Temperature and humidity of the measurement room were maintained at 23.0 ± 1.0°C and 50.0 ± 7.0% throughout the experiments, respectively. All the measurements were done 2 hours after intaking 400 kcal carbohydrate as lunch. Participants were requested not to smoke and not to expose themselves to high physical activity before exercise. Body weight, height and lean body mass were measured using a body fat analyzer (TANITA, BC-522, Japan), while body mass index (BMI) was calculated as weight (kg) divided by squared height (m^2^). By using the Karvonen method, the maximal heart rate of the each participant was evaluated and the target heart rate during exercise was determined. Energy expenditure was measured in the sitting position for 30 minutes both at rest and during exercise. The subjects performed an aerobic exercise program with a bicycle ergometer (75XL2ME, COMBI WELLNESS, Japan) at 60% of their maximal heart rate for 30 minutes, and the pedaling frequency was set at 60 rpm. Oxygen consumption (V02), carbon dioxide production (VCO2), and respiratory quotient (RQ) were measured using a whole-body indirect human calorimeter (AE300S, MINATO Medical Science, Japan). Energy expenditure (EE), fat oxidation (FO), and carbohydrate oxidation (CO) at rest and during the exercise were calculated from RQ by using the Lusk table. All data were expressed as kilocalories per 30 minutes.

### 2.4. Statistical Analysis

Statistical analysis was performed using the Statcel97 PC software (OMS, Japan). All data are presented as the mean ± SD. Statistical significance of the differences between the genotypes was evaluated with the ANOVA (followed by post hoc Bonferroni tests) or Student's *t*-test, if needed. Statistical significance was set at *P* < .05.

## 3. Results

The mean age and BMI of 86 college students (45 males, 41 females) recruited for this study were 22.2 ± 3.6 years and 22.6 ± 3.4 kg/m^2^, respectively. The BMI of the males and the females were 23.1 ± 3.6 kg/m^2 ^and 22.2 ± 3.1 kg/m^2^, respectively. 

Genotyping of the Trp64Arg polymorphism in the *β*3-AR gene revealed that the distribution of the Trp/Trp, Trp/Arg, and Arg/Arg genotypes was 52 (60.5%), 33 (38.4%), and 1 (1.1%), respectively. The allele frequency of the Arg64 was 20.3%. The subjects were separated into two groups, Trp/Trp and Trp/Arg + Arg/Arg, to analyze the association of the genotype with energy expenditure, because the number of subjects with the Arg/Arg was too small for statistical analysis. 

Table 1 shows the physical characteristics and the energy expenditure of the subjects with Trp/Trp and with Trp/Arg + Arg/Arg. In the male subjects, there was no significant difference in the resting energy expenditure (REE_0–30_) between the two groups, whereas it was demonstrated that the subjects with the Arg allele showed the higher level of resting carbohydrate oxidation (RCO_0–30_) and the lower level of resting fat oxidation (RFO_0–30_). Energy expenditure during exercise (EEE_0–30_) in subjects with the Arg allele was comparable to that without the Arg allele. There was, however, difference in the energy source during exercise, as at rest. The level of fat oxidation (EFO_0–30_) in the subjects without the Arg allele was significantly higher than that in those with the Arg allele, though there was no difference in the level of carbohydrate oxidation (ECO_0–30_). 

Analysis of the female subjects also revealed that no significant differences were observed in the REE_0–30_ and EEE_0–30_ between the subjects with and without the Arg64 allele, unlike in the male. In contrast to the male, there were not any differences in the fat oxidation and the carbohydrate oxidation both at rest and during exercise between the Trp/Trp and the Trp/Arg + Arg/Arg. 

The distribution of A/A, A/G, and G/G in the −3826A/G polymorphism of UCP1 gene was 19 (22.1%), 44 (51.2%), and 23 (26.7%), respectively. The physical characteristics of this polymorphism in the male and in the female and the energy expenditure in the subjects with the A/A, A/G, and G/G genotypes were shown in Table 2. In the resting state, there were not any substantial differences in the REE_0–30_, RFO_0–30_, and RCO_0–30_ among the subjects with A/A, A/G, and G/G both in the male and the female. During aerobic exercise, the EE_0–30_, FO_0–30_, and CO_0–30_ in the male subjects with G/G seemed to be lower than those of A/A (*P* = .13, .38, .09, resp.). Analysis in the female subjects revealed that there were not any substantial differences in the EE_0–30_, FO_0–30_, and CO_0–30_ among the subjects with A/A, A/G, and G/G. 

To elucidate whether *β*3-AR Arg64 and UCP1 –3826G alleles affected synergically to lower the energy expenditure both at rest and during exercise, the subjects were regrouped into four categories according to their *β*3-AR and UCP1 genotypes: (group 1) subjects with Trp/Trp of the *β*3-AR gene and A/A in the −3826A/G polymorphism of UCP1 gene (n: 5 males, 8 females), (group 2) subjects with Trp/Arg or Arg/Arg of the *β*3-AR gene and A/A in the −3826A/G polymorphism of UCP1 gene (n: 4 males, 2 females), (group 3) subjects with Trp/Trp of the *β*3-AR gene and A/G or G/G polymorphism in the −3826A/G of UCP1 gene (n: 24 males, 15 females), and (group 4) subjects with Trp/Arg or Arg/Arg of the *β*3-AR gene and A/G or G/G polymorphism in the −3826A/G of UCP1 gene (n: 12 males, 16 females). 

In the resting state, though significant differences could not be detected because of the small subject size, the male subjects in group 4 had relatively lower RFO_0–30_ (0.23 ± 0.16 kcal/kg versus 0.40 ± 0.28 kcal/kg, *P* = .07) and higher RCO_0–30_ (0.45 ± 0.11 kcal/kg versus 0.30 ± 0.22 kcal/kg, *P* = .07) compared with the subjects in group 1. There were not any significant differences in the REE_0–30_, RFO_0–30_, and RCO_0–30_ of the males and females among the groups. During the exercise, no significant differences could be detected in EEE_0–30_, EFO_0–30_, and ECO_0–30_ of the male and female subjects. 

## 4. Discussion

This study revealed that the Trp64Arg polymorphism in the *β*3-AR gene is associated with reduction of fat oxidation not only at rest but also during aerobic exercise in the male subjects of healthy young Japanese. As the polymorphism did not affect the level of energy expenditure, the present data would imply that exercise for a long duration is necessary to utilize the energy stored as lipids for male individuals with the Arg64 allele in the *β*3-AR gene, compared with the individuals without the Arg64 allele. 

Aerobic exercise in healthy subjects was reported to increase lipid oxidization during exercise by elevating circulating cathecholamine level, which facilitated lipolysis, in the white adipose tissue through *β*3-AR signaling [14]. The increased lipolysis raised the free fatty acid (FFA) level and delivered energy to skeletal muscles for fat oxidation [15]. In subjects with Trp to Arg substitution at codon 64 of *β*3-AR gene, intracellular cAMP level after the stimulation with cathecholamine is documented to be lower than that without the substitution, which results in attenuated hormone-sensitive lipoprotein lipase activity, lipolysis and thermogenesis [16]. 

Difference was observed between the males and the females with Arg64 allele in the lipolytic activity during aerobic exercise. This might be explained by the different distribution of visceral and subcutaneous fat in both sexes. Males accumulate fat in the abdominal region through the action of testosterone, whereas the female in the gluteal-femoral regions by that of estrogen [17]. It is well known that lipids stored in the visceral adipose tissue are consumed rapidly during aerobic exercise, compared with those stored in the subcutaneous adipose tissue [18]. As it is shown that the visceral adipocytes express the *β 3*-AR gene four times more abundantly than the subcutaneous fat [19], the fat oxidation level of the male is thought to be highly influenced by the presence of Trp to Arg substitution at codon 64 of *β 3*-AR gene. The fat oxidation level during aerobic exercise in the female subjects may be influenced by this substitution, though a little, because of the smaller volume of the visceral fat. 

UCP1 is present exclusively in brown adipose tissue and plays a significant role in the control of energy expenditure [7]. Several recent studies have shown that *β*3-adrenoreceptor agonists and some dietary constituents induce expression of UCP1 not only in the brown adipose tissue but also in the white adipose tissue [20, 21]. Although UCP1 expression in the white adipose tissue leads to an increase in energy expenditure via the generation of heat, it is clear that UCP1 is involved in cold-induced nonshivering and diet-induced thermogenesis [7], but not in the exercise. As UCP1 does not play an important role in the energy expenditure during aerobic exercise, the effects of the A to G substitution in the UCP1 gene on fat oxidation were not confirmed in the present study. Our study demonstrated the importance of *β*3-AR Trp64Arg polymorphisms in fat oxidation during aerobic exercise in the male Japanese subjects. 

Although Valve et al. reported the synergic effect of the two gene polymorphisms on basal metabolic rate in Finns [5], the effect of the two gene polymorphisms on energy expenditure at rest or during exercise was not detected in the present study. We could not exclude the possibility, however, that the number of subjects in each group was too small to detect significant differences, because the primary purpose of our study was to evaluate whether these two gene polymorphisms independently affected the energy expenditure during exercise. Further studies would be needed to clarify whether presence of the both polymorphisms in the *β*3-AR gene and UCP1 gene would act synergically on energy expenditure and fat oxidation during exercise, as on energy expenditure at rest. 

Our study would suggest the necessity of an individualized menu of aerobic exercise for type 2 diabetics for efficient outcome according to their genotypes in *β*3-AR. However, further investigations are needed to fully elucidate the effect of the *β*3-AR Trp64Arg polymorphism on fat oxidation during aerobic exercise in patients with type 2 diabetes. It was reported that aerobic training in type 2 diabetes or obese subjects increased fat oxidation during exercise [21]. As our study was performed on young healthy Japanese subjects, it is unclear whether the effect of the physical training in patients with type 2 diabetes is modified by the *β*3-AR Trp64Arg polymorphism similarly to that in the healthy subjects. 

Though long-term regular physical exercise has been reported to increase mitochondrial enzyme capacity, total mitochondrial volume, and fat oxidation [22], it also remains unclear whether the *β*3-AR Trp64Arg polymorphism influences mitochondrial functions and fat oxidation after long-term aerobic training. As there have been no observations about the effect on *β*3-AR Trp64Arg polymorphism and fat oxidation, a long-term prospective study is expected to learn the necessity of individualization of aerobic exercise in strength and length in patients with diabetes. 

In summary, it was revealed that the Trp64Arg polymorphism in *β*3-AR gene was associated with reduction of fat oxidation both in resting and aerobic exercise in healthy, young Japanese males. Healthy young Japanese males with the Arg64 allele should perform exercise for longer periods than those with the Trp64 allele to the reduction of fat oxidation.

## Figures and Tables

**Table 1:** The physical characteristics and energy expenditure in subjects with the *β*3-AR Trp64Arg polymorphism. Data are expressed as means ± SD. *P < .05.

	Male			Female		
	Trp/Trp	Trp/Arg	*P*	Trp/Trp	Trp/Arg	*P*
		Arg/Arg			Arg/Arg	
Number of subjects	29 64.4%	16 35.6%		23 56.0%	18 44.0%	
Age (years)	21.6 ± 2.3	23.1 ± 3.9	0.16	21.6 ± 2.8	22.7 ± 5.3	0.42
Height (cm)	172.7 ± 5.1	171.5 ± 6.8	0.55	158.3 ± 5.1	159.8 ± 6.5	0.39
Weight (kg)	68.7 ± 9.3	68.3 ± 15.2	0.90	56.8 ± 7.3	53.7 ± 7.5	0.18
BMI (kg/m^2^	23.0 ± 2.7	23.2 ± 5.0	0.87	22.7 ± 3.2	21.0 ± 2.5	0.06
Lean body mass (kg)	52.3 ± 4.6	51.1 ± 6.5	0.47	37.4 ± 5.3	35.6 ± 2.7	0.21
In the resting state						
(kcal/kg/30min)						
Energy expenditure	0.72 ± 0.13	0.69 ± 0.11	0.47	0.69 ± 0.11	0.70 ± 0.14	0.47
Fat oxidation	0.38 ± 0.18	0.23 ± 0.16	*0.01	0.38 ± 0.18	0.32 ± 0.15	0.29
Carbohydrate oxidation	0.34 ± 0.17	0.46 ± 0.12	*0.01	0.31 ± 0.18	0.38 ± 0.14	0.20
During the aerobic exercise						
(kcal/kg/30min)						
Energy expenditure	3.12 ± 0.48	3.07 ± 0.44	0.74	2.24 ± 0.33	2.34 ± 0.27	0.33
Fat oxidation	1.00 ± 0.38	0.78 ± 0.28	*0.05	0.74 ± 0.29	0.76 ± 0.32	0.89
Carbohydrate oxidation	2.12 ± 0.44	2.29 ± 0.38	0.22	1.50 ± 0.41	1.58 ± 0.36	0.48

**Table 2:** The physical characteristics and energy expenditure in subjects with the UCP1-3826A/G polymorphism. Data are expressed as means ± SD. *P < .05.

	Male				Female			
	A/A	A/G	G/G	*P*	A/A	A/G	G/G	*P*
Number of subjects	9 (20%)	27 (60%)	9 (20%)		10 (24.4%)	17 (41.5%)	14 (34.1%)	
Age (years)	* *24.1 ± 4.7	* *21.6 ± 2.4	* *21.7 ± 2.0	0.10	* *20.8 ± 0.6	* *23.1 ± 6.0	* *21.9 ± 2.0	0.36
Height (cm)	* *169.2 ± 7.4	* *172.8 ± 5.4	* *173.7 ± 3.5	0.17	* *156.9 ± 5.4	* *160.1 ± 6.8	* *159.1 ± 4.4	0.37
Weight (kg)	* *63.7 ± 10.1	* *67.6 ± 8.6	* *76.3 ± 17.1	*0.05	* *58.5 ± 7.8	* *55.1 ± 7.5	* *53.7 ± 7.0	0.30
BMI (kg/m^2^)	* *22.2 ± 3.0	* *22.6 ± 2.8	* *25.2 ± 5.6	0.13	* *23.8 ± 3.9	* *21.4 ± 2.5	* *21.2 ± 2.6	0.07
Lean body mass (kg)	* *49.2 ± 5.03	* *51.7 ± 4.47	* *55.2 ± 6.31	*0.04	* *37.5 ± 5.31	* *36.7 ± 5.28	* *35.6 ± 2.34	0.57
In the resting state								
(kcal/kg/30min)								
Energy expenditure	* *0.73 ± 0.07	* *0.70 ± 0.13	* *0.72 ± 0.11	0.82	* *0.68 ± 0.15	* *0.69 ± 0.08	* *0.71 ± 0.15	0.8
Fat oxidation	* *0.35 ± 0.22	* *0.34 ± 0.16	* *0.25 ± 0.24	0.43	* *0.38 ± 0.18	* *0.36 ± 0.17	* *0.32 ± 0.16	0.65
Carbohydrate oxidation	* *0.39 ± 0.20	* *0.36 ± 0.15	* *0.47 ± 0.16	0.26	* *0.29 ± 0.17	* *0.33 ± 0.15	* *0.39 ± 0.21	0.42
During the aerobic exercise								
(kcal/kg/30min)								
Energy expenditure	* *3.38 ± 0.69	* *3.06 ± 0.33	* *2.98 ± 0.50	0.13	* *2.29 ± 0.37	* *2.33 ± 0.34	* *2.25 ± 0.21	0.78
Fat oxidation	* *0.95 ± 0.44	* *0.97 ± 0.35	* *0.78 ± 0.29	0.38	* *0.69 ± 0.34	* *0.85 ± 0.27	* *0.68 ± 0.30	0.23
Carbohydrate oxidation	* *2.43 ± 0.40	* *2.09 ± 0.42	* *2.20 ± 0.30	0.09	* *1.60 ± 0.51	* *1.48 ± 0.35	* *1.57 ± 0.34	0.70
